# “In the office nine to five, five days a week… those days are gone”: qualitative exploration of diplomatic personnel’s experiences of remote working during the COVID-19 pandemic

**DOI:** 10.1186/s40359-022-00970-x

**Published:** 2022-11-17

**Authors:** Samantha K. Brooks, Charlotte E. Hall, Dipti Patel, Neil Greenberg

**Affiliations:** 1 Department of Psychological Medicine, King’s College London, Weston Education Centre, London, SE5 9RJ UK; 2 Behavioural Science and Insights Unit, Emergency Response Department Science and Technology, UK Health Security Agency, Porton Down, Salisbury, SP4 0JG UK; 3 Foreign, Commonwealth and Development Office, King Charles Street, London, SW1A 2AH UK

**Keywords:** COVID-19, Diplomatic personnel, Diplomats, Pandemic, Remote working, Working from home

## Abstract

**Background:**

Many employees had to work remotely during the COVID-19 pandemic. Literature suggests there are both challenges and benefits to remote working and that remote working can have detrimental effects on mental health. This study aimed to explore diplomatic personnel’s perceptions and experiences of working from home during the pandemic.

**Methods:**

Twenty-five employees of the Foreign, Commonwealth and Development Office took part in semi-structured interviews. Thematic analysis was carried out to extract recurring themes from the data.

**Results:**

Seven main themes emerged from the data: impact of the pandemic on work; relationships with colleagues; benefits of working from home; challenges of working from home; family; moving posts during the pandemic; and perceptions and predictions of post-pandemic work. Participants provided mixed views on how remote working had affected productivity and relationships with colleagues. Benefits of working from home included greater freedom and flexibility; new opportunities; and inclusivity of remote meetings. Challenges included being in different time zones to the countries they were working for; unsuitable home ergonomics; technological issues; and difficulties finding appropriate work-life balance. Those with young children reported difficulties juggling work and childcare. Adjusting to new posts at a time when staff were working remotely appeared particularly challenging. However, most did not want or expect to return to entirely office-based work. They predicted a hybrid model of working in the future, involving both office work and remote work; they stressed the importance of flexibility and suggested there would not be a one-size-fits-all approach to returning to face-to-face work.

**Conclusions:**

Remote working during the COVID-19 pandemic has changed the ways in which employees work, showing them that they do not have to be in the office to successfully achieve their work goals and leaving many wanting flexibility to make their own decisions about working from home (or not). There are both benefits and challenges to remote working; managers can take steps to reduce some of the challenges by being available to support their employees, organising regular remote meetings and allowing employees autonomy in terms of when and where they work.

**Supplementary Information:**

The online version contains supplementary material available at 10.1186/s40359-022-00970-x.

## Background

December 2019 saw the outbreak of novel coronavirus COVID-19 which spread rapidly, leading the World Health Organization (WHO) to declare a global pandemic on March 11^th^ 2020 [1]. COVID-19 soon became an unprecedented global crisis which forced people to restrict their social contact and changed the ways in which people lived and worked. As a result, over half of those in employment in the United Kingdom (UK) reported working from home in April of 2020 [2]—a substantial increase from the 5% estimated to work from home pre-pandemic [3]. Despite pandemic restrictions easing, the percentage of people working from home since 2020 remains high [4].

Literature on homeworking pre-pandemic has reported many benefits, such as eliminating commuting time and thus providing more time for relaxing or other responsibilities [5], but also challenges such as a sense of blurred boundaries between work and home life [6]. Literature on the mental health impact of the COVID-19 pandemic on employees suggests that poor home ergonomics [7], difficulties with using the technology required for remote working [8] and work-life conflict [8] can all contribute to poor mental health and wellbeing. Indeed, working from home during the COVID-19 pandemic has been shown to have a negative impact on both mental and physical health [9]. Although there is to date little literature on remote working for employees working outside of their home countries, it has been suggested that international employees may have a particularly difficult time working effectively during the pandemic, having to cope with the uncertainty and unfamiliarity of working during a global pandemic at the same time as adjusting to a new country and culture [10] as well as worrying about both local transmissions and the risk to families in their home countries [11].

One occupational group who frequently work overseas is diplomatic personnel. Relatively little is known about the work-related wellbeing or work satisfaction of this group [12] and to date there has been no published empirical research exploring the lived experiences of diplomatic personnel during the pandemic. It is likely that the emergency response to the pandemic—including the closing of borders, cancelling of international visits and various social restrictions put in place, including strict public health interventions in some countries—has had a direct impact on diplomacy, including diplomatic interactions and communication, and diplomats’ adjustment to their host country [13, 14]. Academic literature also acknowledges that diplomats’ roles may also have changed to include monitoring COVID-19 information and engaging with the public in different ways, and that this shift in work will require rapid adaptation [14–16]. However, the impact of remote working and the various potential benefits and challenges of remote working for diplomatic personnel have not yet been explored. To fill this gap in the literature, this study aimed to gather qualitative data from diplomatic personnel relating to their experiences of remote working during the COVID-19 pandemic.

## Methods

### Design

This qualitative study used a semi-structured interview design—that is, interviews involved a series of questions asked to all participants, but were flexible in terms of having open-ended questions; allowing the interviewer freedom to ask participants to elaborate on particular answers; and allowing the participant to direct the flow of the interview [17]. Thematic analysis was used to analyse the data and one author (SKB) was responsible for the coding of the interview transcripts.

### Participants

Participants were employees of the Foreign, Commonwealth and Development Office (FCDO), a UK government department responsible for safeguarding the UK’s national security, managing international relations, tackling global challenges with international partners, and supporting British nationals around the world. To be eligible for inclusion in the study, participants had to be aged 18 or over; currently employed by the FCDO; and employed by the FCDO for at least six months.

### Procedure

An invitation letter outlining the nature and aims of the study and providing the researchers’ contact details was sent to welfare staff at the FCDO, who emailed the invitation to 100 randomly selected diplomatic staff who met the inclusion criteria. This invitation letter asked potential participants to email the researchers of their own volition if they wanted to volunteer to take part or request further information. Due to a low response rate to the first invitation, two further rounds of invitations were sent to a random selection of staff in a variety of posts. We requested that if the welfare office perceived that particular staff members might experience significant distress as a result of the study, those staff should be excluded from the invite list. Those who were interested in taking part emailed the researchers directly, at which point full Information Sheets and Consent Forms were sent to participants and interviews were arranged. Interviews were carried out by one researcher (SKB) between September 2021 and February 2022. All interviews were carried out using the online audio-conferencing platform Microsoft Teams, with the exception of one which began on Teams and moved to a telephone call due to poor internet connection.

### Interviews

An interview guide was developed by SKB and NG with input from FCDO staff, which included central questions to be asked in each interview. As this was part of a wider study on diplomats’ experiences of the COVID-19 pandemic, a broad range of questions relating to potential stressors (both in and outside of the workplace) and perceptions of organisational support were asked; the questions relevant for this particular study related to whether or not participants had worked remotely during the pandemic, their experiences (including perceived benefits and challenges) of working from home, and their perceptions of whether remote working would continue beyond the pandemic. Interviews lasted between 23 min 18 s and 57 min 58 s (median: 33 min 41 s). Interviews were recorded and transcribed verbatim by SKB.

### Analysis

Transcripts were stored and coded on NVivo software [18]. Data were analysed inductively by one author (SKB) according to the six-stage approach to thematic analysis [19]. This approach involved, firstly, familiarisation with transcripts: each transcript was read thoroughly several times in order for the author to familiarise themselves with the content of them. Secondly, line-by-line coding was done to break the data into discrete excerpts, with codes created to summarise the content of each excerpt. The third step involved collating codes into over-arching themes; codes similar to each other in content were grouped together and NVivo tree maps were used to explore patterns in the data. Next, the author reviewed themes to ensure that the data within each theme did fit into the theme, and that the themes appropriately reflected the data corpus as a whole. Close examination of the data within each theme then allowed for the defining and naming of themes. The final stage involved choosing quotes to appropriately illustrate the themes in the write-up. The remaining three authors read the list of themes and the quotes chosen to ensure that they were representative of each theme and that the structure of the analysis was logical.

### Ethics

The study was carried out in accordance with the British Psychological Society Code of Ethics and Conduct [20] and the General Data Protection Regulation 2018 [21]. Participation was entirely voluntary and there were no consequences for declining to take part. All participants received Information Sheets and signed an Informed Consent form prior to participation. All were reassured of their right to withdraw at any time and that no identifying details would be shared. All participants were also reassured that the FCDO would not know which individual members of staff took part. The research was approved by the Psychiatry, Nursing and Midwifery Research Ethics Subcommittee at King’s College London (ethical clearance reference number: HR/DP-20/21-22511).

### Reflexivity

The researcher continually reflected on how their own experiences or expectations may have influenced their interactions with participants or interpretation of the data. Immediately after each interview, overall thoughts on the interview process were recorded in NVivo, allowing the interviewer to reflect on their interview technique and consider whether any questions could be improved as well as allowing the interviewer to reflect on their role in the data collection process. Although the interviewer may have had their own assumptions prior to doing this study, throughout the interviews they consciously questioned these assumptions and encouraged participants to talk freely about their own thoughts and experiences. Follow-up probing questions were used throughout the interviews to clarify that the interviewer had understood responses.

## Results

A total of 46 FCDO employees contacted the researchers for further information about the study and 25 agreed to take part in the research and subsequently participated in interviews. As some participants reported sharing the study invitation with colleagues, it is not possible to know exactly how many individuals received the study information and consequently not possible to calculate an overall response rate; of the 46 who contacted the researchers, 54.3% subsequently took part. The sample included 14 (56%) males and 11 (44%) females, with ages ranging from early 20s to mid-60s (mean 46 years). Time with the organisation ranged from less than one year to over thirty years (mean: 13 years 8 months, median: 13 years), and participants worked in a variety of roles and a wide range of grades within the FCDO. Most (92%) had worked overseas for at least some time during the pandemic. Participants had been based in 23 different countries across six continents since the COVID-19 pandemic began; Table 1 provides detail on geographical distribution. The classification of continents and regions presented are based on the United Nations geoscheme [22] with the United Kingdom presented separately due to this being of particular relevance to this study (i.e. participants based in the United Kingdom were working in their home country). Two participants had worked in fragile state posts for at least some of the time during the pandemic whilst 17 reported having deployed to ‘hardship locations’ (locations with extremely difficult living conditions) where they received hardship allowance.

**Table 1:** Geographical distribution of participants

Continent/region	Number of countries represented	Number of participants*
*Europe*		
United Kingdom	1	7
Southern Europe	1	1
Western Europe	1	1
*Americas*		
Northern America	1	6
Central America	2	2
South America	3	3
Oceania	1	1
*Africa*		
Northern Africa	4	4
Southern Africa	1	2
Eastern Africa	2	2
*Asia*		
Central Asia	1	2
Southern Asia	2	2
East Asia	1	1
Western Asia	2	5

^*^Note that the sum total of participants representing different countries is greater than the number of participants in the study; this is due to several participants relocating at least once during the pandemic

Table 2 provides an overview of the themes and sub-themes which emerged from the data. Additional file 1: Appendix 1 illustrates each theme/sub-theme with relevant quotes.

**Table 2:** Themes and sub-themes

Theme	Sub-themes
Impact of the pandemic on work	Pre-COVID experiences of remote working; Changes to working style; Productivity; Privacy and security
Relationships with colleagues	Sense of community; Reduced interaction with colleagues; Being away from negative relationships
Benefits of working from home	Freedom and flexibility; New opportunities; Inclusivity
Challenges of working from home	Time zones; Home ergonomics; Reliance on screens and technology; Work-life balance
Family	Improved relationships; Childcare
Moving posts during the pandemic	Adjusting to new posts; Lack of social events; Language barriers; Leaving old posts
Perceptions and predictions of post-pandemic work	New views on remote working; Benefits of returning to face-to-face working; Challenges of returning to face-to-face working; Hybrid working; Considerations for the future

Participants were given a unique identification code (P1 through to P25) to protect their anonymity. An ellipsis in brackets—‘(…)’*—*within a quote indicates the removal of text (for example, conversational fillers or responses where the removal of words did not change the meaning of what was said), and text in square brackets—‘[Text]’*—*within a quote indicates text inserted by the author for clarification.

### Impact of the pandemic on work

#### Pre-COVID experience of remote working

Most participants had never, or very rarely, worked remotely until the pandemic. Several suggested that, until the pandemic, working from home had never really been a consideration for them as it was simply presumed to be *“not really feasible in my line of work” (P3).* Some also described the FCDO as having been ‘behind’ other organisations in terms of their (lack of) ability to work from home prior to the pandemic. Only one reported having frequently worked remotely before the pandemic, and they felt their experiences had been useful in terms of showing the organisation that working from home was possible.

#### Changes to working style

All participants reported that the pandemic had altered their ways of working. The majority (23/25) had worked from home at least some of the time during the pandemic; the remaining two participants reported going into their embassies throughout the pandemic, but as so few others were going into the office at all, their meetings were all carried out remotely. Some remained overseas but worked from their homes there, whereas others were forced to return to the UK or were ‘stuck’ in countries other than the ones they were officially working for due to being unable to travel. For many, their specific duties did not change but the way they went about them did, with participants suggesting *“you had to find different ways to be able to continue doing what you were doing” (P8).* Most reported that the biggest change was the sudden reduction of in-person interactions on which their roles had always relied.

#### Productivity

Some participants preferred working from home as they could focus and concentrate more easily without distractions from colleagues, suggesting that *“the team is so big and when you're in the office, it's very, very noisy and it's quite hard to concentrate so I think we've all found that a bit of a blessing as well, being able to work from home (…) it's much, much easier to concentrate” (P11).* This was particularly helpful if they were involved in particularly difficult or time-consuming projects. Conversely, a minority felt they were less productive when left to their own devices and not around their colleagues or reported that the inability to have spontaneous in-person discussions with people meant that their work was slower. These mixed results relating to the perceived impact of remote working on productivity suggest that whether productivity is helped or hindered by working away from colleagues is dependent on the individual and their own preferences and working style.

#### Privacy and security

Working from home was frequently described as beneficial for those whose work involved lots of confidential telephone discussions, as it meant they could have those conversations without worrying about others overhearing. Conversely, others felt unable to do their job properly from home because they felt they lacked the technological security they needed.

### Relationships with colleagues

#### Sense of community

For some participants, holding meetings remotely bolstered the sense of community within the organisation because it allowed them to connect with people they would not usually be seeing. Others reported successfully using online platforms to do virtual ‘social events’ with colleagues which helped foster a sense of community within teams*.* One participant, who had moved posts during the pandemic, suggested that the use of Microsoft Teams to interact with people helped them get a better sense of their new network because they could ‘meet’ remotely with people they would not have been meeting face-to-face. Even participants who were working with their usual teams sometimes reported that remote meetings allowed them to get to know their colleagues better than in-person meetings, with virtual meetings providing glimpses into employees’ personal lives and allowing for better understanding of what their lives outside of work involved:

People aren’t physically together (…) but at the same time you do get a lot more insights into individuals’ personalities (…) you have a meeting and suddenly you see where [they live], you see their kids, you see their pets, you know the real kind of personalities and people talk more about it because that line has been blurred and we have to really understand a lot better individual circumstance to ensure that people can do their jobs and juggle everything else that’s going on in their lives, so that’s been a positive thing in a way, that you really get to know each other a lot better, understand how we tick and what’s important to us (P4).

Another reason for the development of stronger relationships with colleagues appeared to be the fact there were fewer opportunities to socialise with others outside the organisation.

#### Reduced interaction with colleagues

A small number of participants did not appear to share these views of an increased sense of community, instead suggesting that they did not interact enough with colleagues when working from home. Some reported missing their friendly interactions with colleagues in the office—*“we just have fun in the office, that's the thing and you miss out on that interaction and that you know singing and dancing and being silly stuff (…) that kind of stuff I miss” (P5)*—whilst others missed networking with those they should be meeting in the countries they were based in: *“I’m having some business meetings online, but it’s not the same, it’s not the same as building that rapport in person, so that’s a big frustration to be honest” (P5).* Some also suggested that not being physically around colleagues had impeded their ability to have more difficult conversations as well as their ability to understand the rationale behind organisational decisions.

#### Being away from negative relationships

A small number of participants reported poor relationships with colleagues or managers, and reported that the ability to work from home eased the stress caused by this.

### Benefits of working from home

#### Freedom and flexibility

Many participants reported that remote working gave them the opportunity to fit day-to-day household tasks in around their work more easily. In particular, participants felt that not having to commute gave them more free time for relaxing, spending time with family, and taking part in hobbies or self-care activities. Some participants suggested that the new-found flexibility resulting from working from home had altered the way they felt about going into work:

I think people kind of see almost the task of going to the office as kind of an arduous thing now which they don’t really enjoy doing necessarily. I think there’s a number of things that people like, like the flexibility they get in their lives from working from home, like you get up late, you don’t need to commute, you don’t need to kind of dress as you would for the office, you don’t need to think about planning your lunch (…) you can go out and exercise or to the shops in the middle of the day (…) all that freedom (P9).

#### New opportunities

Some participants suggested that the shift to remote working had allowed them to develop new skills, such as how to manage others when working remotely. Others found that remote meetings created new business opportunities, allowing them to engage with individuals and companies they would not otherwise have met. The fact that employees were working from home, with missions empty, also provided opportunities for renovation, which would have been much for challenging if the buildings were full.

#### Inclusivity

The shift to remote working was seen as a benefit for members of staff who were perhaps more introverted or uncomfortable with in-person meetings, or had neurodiverse conditions causing them to struggle with eye contact and face-to-face interactions, allowing them a safe space to participate and develop their confidence.

### Challenges of working from home

#### Time zones

Due to government restrictions on travel, several participants were in different countries and even different continents to those they were working for. One particular challenge noted by participants forced to work from countries other than the ones they were supposed to be in was the difficulty of managing the time difference, with participants often having to get up very early and work odd hours in order to have remote meetings with people in other countries.

#### Home ergonomics

Some participants felt they had unsuitable home ergonomics for working remotely, with lack of space and suitable workspaces often leading to discomfort and pain: *“my back was killing me 'cause I didn't have an office or anything, sitting on the dining room chair just didn't work” (P22).* Some who were unable to travel to their overseas accommodations were forced to work from hotels, Airbnbs or holiday homes in the UK which lacked suitable work-space.

#### Reliance on screens and technology

Some participants were tired of having to spend all day on their computers and in remote meetings: *“people have just got fed up of sitting looking at a screen all day” (P5).* Additionally, some reported challenges with using the technology required for remote meetings, such as attendees frequently forgetting to unmute themselves in meetings.

#### Work-life balance

Some participants reported that it was harder to find an appropriate work-life balance when working from home: *“That kind of work life balance split, like not really feeling the kind of switch-off in the evening (…) some of that I found that quite challenging” (P3).* Those who lived on compounds with their colleagues reported particular difficulties finding an appropriate work-life balance because they felt unable to escape from their working life.

### Family

#### Improved relationships

Many participants reported that the pandemic had allowed them more time for connecting with friends and family. Some felt that relationships had strengthened: *“doing the Zoom calls and stuff with school friends and things you know, we’ll probably make more of an effort now to have reunions and things (…) things like that have been better” (P5).* Participants working in the UK contrasted the amount of time they could spend with family during the pandemic to how little time they spent together usually. Many participants reported liking the fact that working from home allowed them to spend more time with partners and children: *“I see a lot more of my children (…) when I work in the office I tend to come home and see them at five fifty and they go to bed at six. Whereas when I'm at home I see them throughout the day” (P18).*

#### Childcare

Several participants had young children who were not yet in school and so they were having to juggle remote working with looking after their children. Nurseries were not always an option, either due to closure because of the pandemic or because participants did not want to start their children on a routine they may have to quickly take them out of again, because they were not sure when they would be able to travel overseas to their new posts. All participants with young children reported finding it difficult to work from home; *“at home trying to do our normal job with young children running around (…) just didn’t work basically, it was incredibly stressful” (P21)*. Even when their partners were able to care for the children, simply being at home with young children who did not understand why one of their parents could not spend time with them was often difficult:

[Young son] didn’t understand that Mummy was at home for so many hours and working because usually I tried to keep a clear divide between work and home (…) he didn't understand why I was in meetings, why my phone was glued to my ear (…) he would, you know, do things to disrupt that, he would take my phone away, switch it off, he would come in with the noisiest toy, switch off the Internet (…) unless I locked myself in the room, he would find a way to come in and disrupt things (P17).

Participants juggling work and childcare frequently spoke of feelings of guilt and shame about not being able to give a hundred per cent to either their work or their children: *“I was enormously stressed (…) ‘cause I just felt like I wasn’t pleasing anybody (…) I was just constantly panicking because I wasn’t doing my job, I wasn’t there, I wasn’t on top of my game like I always had been (…) so I was very stressed, very anxious, just feeling like I wasn’t pleasing anyone at all and always felt like people were judging me, even though I’m sure they weren’t” (P21).* Having managers who also had children was reported to be beneficial, as they were perceived to understand the struggles involved, whereas those without childcare responsibilities were perceived to find it harder to understand. The perception that others who were not in the same situation would not be able to understand their struggles appeared to cause substantial distress and led participants to feel they were being judged by their colleagues for not being able to work to their usual standards or work their usual hours. Other stressors relating to childcare included needing to work odd hours to fit everything in and struggling to cope with having to take more of a backseat at work.

### Moving posts during the pandemic

Diplomatic personnel are a somewhat unique occupational group in that they move posts frequently, often relocating to entirely new countries every few years. Approximately half of the participants (12/25) had left old postings and started new ones during the pandemic, and they cited various challenges of working from home whilst adjusting to a new post.

#### Adjusting to new posts

Some had already had the chance to visit their new countries or meet their new colleagues prior to the pandemic, and they were thankful for this, acknowledging that starting their new posts would have been more difficult without having had that opportunity. However, many participants who arrived at new posts in new countries during the pandemic had never visited that country before and therefore did not know anyone there: *“being in a foreign country on your own with no support network (…) it was kind of the same for everyone but I suppose it’s worse for us because especially if you start a new job [during] COVID, you know absolutely nobody in that place” (P16).* Due to countries being in lockdown, participants were unable to get to know people in their new countries which appeared to have a negative effect on their wellbeing. Some participants questioned the rationale behind why they had had to move to a new country when they would be working remotely anyway and unable to meet anyone: *“I slightly questioned why we're sending people out to posts when everyone’s working from home (…) it actually probably makes sense for you to stay in the UK, where you've got your support network et cetera and then as and when people are going back to the office you go back to the office so that you're not kind of parachuted into a country on your own” (P16).* Meanwhile, another who had stayed in the UK as they could not travel to their new post remarked on the perceived absurdity of being an ambassador for a country they were not physically in: *“fundamentally, my job is meant to be out and about meeting people, and I couldn't do any of that. I mean, my entire job is to know the political picture in [Southern African country], and I was sitting in a bedroom in [UK county]” (P18).*

#### Lack of social events

One of the most challenging things about starting a new overseas post during the pandemic was being unable to meet with colleagues in person; *“I found it quite difficult when I arrived (…) not having met anybody in real life and having to do almost everything, at least for the first two or three weeks, virtually was very limiting” (P20).* Participants reported being unable to attend the usual receptions and welcome events that they would typically be going to when joining new posts outside of the pandemic. Such events tended to help staff settle into their new countries and roles, and allowed them to get to know people, which was extremely important when moving to a new country where they had no existing support network. One participant had joined their first post with the FCDO during the pandemic and they reported similar concerns about not meeting their colleagues face-to-face, as well as additional challenges with understanding the FCDO as an organisation:

I found it quite hard when I was coming into a new organisation to understand how the FCDO culturally works, to understand how the mechanics work (…) I like the process of walking the floorboards (…) to then be able to meet as many people as possible and to actually see them face to face first so you can then hopefully build a rapport (…) and not trying to do it through the medium of Teams and through online and everything else which I don't think is always as effective (P24).

#### Language barriers

One participant felt that only being able to meet with people remotely exaggerated the language barrier, suggesting it was much harder to speak a different language to others through a screen (e.g. on a Zoom call) than it was to speak to natives in person.

#### Leaving old posts

As well as difficulties with starting new posts, some participants also spoke of the challenges of leaving old posts during periods of lockdown. For example, one participant described their disappointment at not being able to say goodbye to people properly and described leaving the post as feeling anti-climactic: *“in those final months, there would have been making farewell calls on people and having lots of diplomatic events like dinners and receptions (…) it was very sad (…) really going out on a low flat note. You couldn’t say goodbye to people that you’ve known and worked with closely for four or five years (…) you’re reduced to saying your goodbyes and having closure on quite important work and relationships just over a telephone call. I think that was one of the toughest parts of it” (P20).* Participants also described a sense of loss at missing out on saying goodbyes to family and friends, as they had been in lockdown prior to starting their new posts.

### Perceptions and predictions of post-pandemic work

Participants provided mixed views about whether they believed the organisation would continue allowing freedom to work from home, insist on them returning to the office, or allow a hybrid model involving both remote working and office-based working; they also described mixed views about what they wanted to happen. Many participants thought they should be allowed to work remotely all the time other than when it literally could not be avoided—*“you know that there may be times when you need to come in and it can't be avoided, but otherwise as long as the outputs are there, knock yourself out, work from home” (P10)—*whereas a minority were keen to see a return to the office: *“you just never know if some colleagues are ever in the office (…) [it’s] generated some resentment from other colleagues who think well, I'm always working and where's this person? (…) we need to get back to some sort of whatever normality is” (P5).* The majority of participants wanted to be able to make their own decisions about if, when and how they would return to the office, and did not want to feel pressured to do so, particularly if they felt vulnerable due to their health; one participant perceived that management had a *“lack of appreciation of hidden reasons that people have for being worried and not wanting to come into the office” (P8).*

#### New views on remote working

Many participants—who, before the pandemic, had very rarely worked from home and often felt they would not be able to—suggested the pandemic had showed that remote working is indeed possible. The opportunity for employees to demonstrate that they could do their work remotely was seen as a benefit to the organisation as a whole; *“[working from home] also had the benefit of breaking down the barriers we’d always had to fight against the senior management team to show that we could still do it remotely, because there was a lot of presenteeism still within the old school (…) they finally got to see that you can make it work (…) which is one thing that I'm really grateful for because it's always been such a battle in the past and trying to show people what you can do [from home] and now they realise that it is a lot more possible than they thought” (P21).* Several participants pointed out that it is only due to modern information technology that remote working is now possible. Many participants suggested that their successful remote working during the pandemic had led to increased flexibility within their jobs. Some felt that, having proved they could successfully work remotely, they should not be forced to return to the office: *“there might be a 60/40 split to start off with, but my question is well, okay, if we can do it sixty what's the point of the forty? (…) what can't be done by me remotely? (…) I've had no need to go into the office [since March 2020] (…) so what would the need be for me to suddenly have to go into the office?” (P10).*

#### Benefits of returning to face-to-face working

A minority of participants were very keen to return to face-to-face meetings, suggesting that remote meetings were no substitute for in-person interactions—for example, *“I’m convinced that trying to do work, particularly our type of work, through virtual means can be done, but it’s a pale shadow of what diplomacy really can achieve when you can meet people and have dinners and receptions and all the other things. I think that human contact is a really important ingredient that’s definitely missing” (P20).* In particular, some participants suggested there was no substitution for the ability to see others’ body language and build rapport face-to-face.

#### Challenges of returning to face-to-face working

However, many participants appeared less keen to return to their offices for day-to-day work, due to the flexibility that remote working gave to their lives and the myriad of other perceived benefits of flexible working. Some questioned why they would need to return to their offices when they had demonstrated their ability to work remotely for the past two years. Others reported that they would never be able to go into their offices five days a week because there simply was not space for them: *“it’s all ‘back to work!’ but we just can’t (…) because we’ve merged as well [with the Department for International Development] (…) there’s just not enough space (…) you see all these headlines at the moment about you know, civil servants need to get back in the office, we physically can’t, that’s the bottom line. There’s just not the room for us all to go back” (P11).* Participants reported ongoing discussions within their teams as to how to manage the lack of office space, suggesting that one solution might be rotating working days with others in their team so that only a few people would be in the office at the same time.

Another perceived challenge to returning to the office was the fear of catching COVID-19. Participants pointed out that the pandemic is not yet over, and that fears of COVID-19 infection may still make people reluctant to go into work: *“you reassure people, everyone is happy to come back and then suddenly you have another curveball like Omicron and that really spooks people again (…) still people are nervous and so headlines like Omicron, and the fear factor, impacts (…) I think as long as we live in a world where that is a possibility, which let's be honest is going to be for the foreseeable future, this [going into the office] is going to remain a challenge” (P4).*

Additionally, returning to the office was reported to be difficult after having spent so long away from it. Participants who had returned to the office reported feeling it was not the same as it was pre-pandemic: *“I was in [the office] the week before last just for a day, which was nice, but it’s really really weird. You just feel like you don’t get anything done in the office anymore” (P11).*

#### Hybrid working

A small number of participants felt that the organisation would eventually insist upon them returning to the office. They suggested this was due to organisational culture—*“success is graded on how many people there are beavering away rather than outputs (…) the civil service owns rather a lot of very expensive buildings and to have all these buildings with no bums on seats, there's going to be a very difficult conversation for somebody to have. So the easy answer is right, everybody back into the office (…) which is sad. It's an old-fashioned style of management which I think the civil service in particular is replete with” (P10).*

However, the majority of participants believed that a hybrid of remote and office working would be adopted by the organisation in the future, now that employees knew that it was possible to get their work done remotely: *“I think we’ll be a lot more flexible. You won't have people in the office nine to five, five days a week (…) those days are gone, I think. But nor will we have people working at home full-time in the way that they had to during the height of COVID” (P22); “Pandora's Box is open now, right? (…) I don't think you can ever go back to 100% office” (P4).* However, participants noted that there would likely be a period of transition which might have challenges, especially if managers pushed their employees to return to the office. One participant suggested that if the organisation continued to be flexible in terms of allowing remote working, they may be more likely to retain staff and improve wellbeing in their workforce: *“I think that's [working from home] something as an office we should be really, really encouraging of because I think it just leads to happier people who are willing and then able to continue staying working for the office, because they could make it work with their personal lives” (P3).*

#### Considerations for the future

Many participants accepted that hybrid working was going to be likely for the foreseeable future, and suggested the organisation should be considering how to manage the potential challenges of that. For example, one participant considered how new starters would integrate into the organisation if they were working remotely a lot of the time: *“I guess that kind of virtual remote way of working is (…) now going to be here to stay, and really thinking about kind of how to create a team spirit (…) particularly for people when they're joining, having not been around before” (P9).* Participants suggested that there should also be flexibilities within hybrid working, dependent on people’s individual circumstances; they suggested that hybrid working might involve not staying in the office all day on the days they went into the office, or it might involve scheduling all of their face-to-face meetings for one particular day per week and working the rest of the time at home.

## Discussion

This study explored diplomatic personnel’s experiences of working remotely during the COVID-19 pandemic by analysing interviews with 25 employees of the FCDO. Key findings were that remote working had both clear benefits and various challenges; that working from home was particularly difficult for those with young children and those starting new posts; and that a ‘hybrid model’ of working (i.e. at home some of the time, in the office some of the time) was both wanted and expected in the future.

Most had rarely or never worked remotely until the pandemic, and suggested they had previously felt their roles could not be done remotely. However, the majority felt that their experiences of working remotely during the pandemic had changed their views and opened up new ways of working. Some participants felt their productivity was greater at home than in the office, whilst others felt less productive at home. Recent literature reports a multitude of individual characteristics and circumstances which influence how productive an individual is when homeworking, including gender [23, 24]; age [24]; field of work [25, 25]; and available workspace [24]. The results of the current study also suggest that personality and preference might play a part, with some participants finding themselves more productive around their colleagues and others finding it easier to focus without any distractions. Overall, it appears that the impact of remote working on work is different for different people and individual employees will have their own preferences and needs.

Participants also provided mixed responses on how remote working had affected their relationships with colleagues, with many suggesting the pandemic had fostered a greater sense of community within teams due to the many remote meetings they held, providing greater insight into colleagues’ lives than in-person meetings might have done. This appears to be a novel finding as most literature to date has concluded that working from home results in reduced communication with colleagues and feelings of isolation [26]. Indeed, other participants in the current study felt that reduced interactions with colleagues and in particular with superiors lowered their morale and hindered their ability to get their work done and understand why certain workplace decisions had been made.

The effect of remote working on both productivity and job satisfaction is, again, likely to depend on various individual characteristics such as personality and personal preferences relating to workplace interactions. Remote working may be particularly challenging for new or less confident employees who need more direction and guidance from superiors, whereas others who are confident in their own abilities to make decisions and get things done may not be affected by the reduced opportunities to interact with managers. Importantly, the impact of remote working on workplace relationships is also likely to depend on managers themselves: managers who make time to support their employees, who encourage employees to ask questions via email and who arrange regular meetings can improve their employees’ perceptions of workplace relationships, whereas managers who are rarely available for remote meetings or who do not respond to requests for help are likely to have employees who feel they cannot work well from home. Indeed, a recent commentary on remote working [27] recommends that managers should be mindful of the potential for loneliness and hold regular virtual meetings with their employers, ensuring that employees are given feedback, assisted with goal-setting, and have access to appropriate resources for support.

Participants named several benefits to remote working, including more freedom and flexibility to fit their personal lives, chores, responsibilities and hobbies around their work; opportunities to develop new skills and relationships; and the ability for employees who are uncomfortable in social situations to join in. A study conducted on a sample of academics also found there to be benefits associated with homeworking which align with these findings [28]. However, there were also many challenges of working from home, including working at unusual hours due to time-zone differences; unsuitable home ergonomics such as a lack of physical space to work in, unsuitable desks or uncomfortable chairs; ‘screen fatigue’ and technological issues; and difficulties maintaining an appropriate work-life balance. Current literature also associates working from home with challenges which align with these findings. These include blurring of boundaries between work and home life, and exhaustion [28]. It has been suggested that cultivating personal space at home in order to work productively when working remotely may be helpful: this might include switching off phones to avoid distractions, working in a quiet room with privacy from family members (if possible) and ensuring that breaks are taken with time for emotionally connecting with others in order to avoid feeling overly isolated [27]. With regard to unsuitable home ergonomics, Geldart [27] suggests that managers may not recognise that some of their employees do not have appropriate home set-ups for working, and recommends that organisations invest time and funding into ensuring all employees have a safe working space at home and that employees themselves advocate for their needs by requesting the resources and materials they need to work safely and comfortably from home.

The participants’ beliefs about working from home are likely to be similar to those found in other organisations which continued to function during the pandemic. Our findings supported existing literature on working from home which suggests the boundaries between work and home can blur due to essentially being ‘at work’ and connected to the workplace at all times [6]. Previous research has associated being unable to unwind and relax during leisure time with negative health outcomes, and suggested ways of facilitating positive work-life balance initiatives such as training employees to break down work into manageable tasks to be completed on a daily basis; training in distraction techniques; establishing clear home/work boundaries; and restricting use of technology away from work [29]. The increase in remote working during the pandemic has renewed many organisations’ interest in ways of maintaining a healthy work-life balance, with recommendations being made such as avoiding answering work-related calls and emails away from work and finding new and relaxing things to focus on during non-work hours [30]. We suggest that line managers could model good practice by ensuring they themselves take regular breaks and try to avoid sending out-of-hours emails where possible. They should encourage their staff to have set times for starting and finishing work and to spend time relaxing or on their own hobbies. We also suggest that line managers should actively encourage their employees to unwind after work in whichever ways are most effective for them—through exercise, hobbies, socialising with friends, spending time with family or practising relaxation techniques. There is unlikely to be a one-size-fits-all approach to unwinding after work, and so a wide range of suggestions should be made. It may be empowering for employees to be able to contribute to suggestions; it could be helpful to carry out a survey asking employees how they decompress from work and maintain a good work-life balance, and publish the responses in a leaflet for staff to give them tips and advice.

Whilst many of our participants felt that remote working, and having more time to spend with families, had strengthened their relationships with others, there were challenges for those with young families who had to work from home with children around. Having to juggle childcare for younger children with work appeared to be a major stressor, creating feelings of anxiety, pressure, and guilt if participants felt they were unable to give either their children or their work the focus they needed. Previous research has established that those with childcare responsibilities tend to be less productive when working from home and experience fewer benefits of working from home compared to their childless counterparts [28, 31]; it might therefore be beneficial for organisations to (where possible) demonstrate understanding of childcare responsibilities, allow staff with young children to delegate work or have longer deadlines, and/or subsidise childcare expenses; it is important for employers to demonstrate equal respect to other caring responsibilities such as looking after elderly or disabled family [28].

Moving posts during the pandemic was typically described as particularly stressful, with participants describing both difficulties adjusting to new roles and countries without being able to get out and meet anyone in person and disappointment at leaving old posts without proper goodbyes. The usual ‘welcome events’ and social networking which would happen at the beginning of a new post were seen as essential in helping staff to settle in to their new countries and new roles and helping them to understand local customs as well as the dynamics of their teams. Therefore, starting new roles at a time of social restrictions and lockdown was described as leaving staff feeling unprepared and unable to integrate appropriately. This is unsurprising, as the role of a diplomat is highly dependent on trust and confidence developed via personal contact, and adjustment to the host country is based on understanding that country and its people, and involves meeting people and exploring the culture [15]. We propose ensuring that those new to posts are given a tour (even if remotely) and introduced to their teams (even if remotely). Some participants questioned why they needed to move out to posts if they were going to be working remotely anyway, whereas others remained in the UK because they could not get to their posts, and struggled with the time difference and with establishing their credibility with their teams when they were so far away. The difference in opinions here highlights the importance of individual choice and autonomy, and suggests that diplomatic, or similar, organisations could benefit from demonstrating flexibility and listening to the wishes of individual employees. For diplomatic personnel starting new posts, regardless of whether they are in the UK or overseas, provision of training to develop virtual collaboration skills may be useful [10]. Organisations could benefit from considering how to facilitate informal support networks and hosting remote introductory events.

Scholars have previously raised concerns about the effect of remote working on diplomats, suggesting that—given the profession is based on frequent, face-to-face meetings and private communications about sensitive issues—diplomats would experience a major shift to their work requiring rapid adaptation [13, 16]. Indeed, our participants described having to adjust their work to remote platforms. Whilst some felt being a ‘virtual’ diplomat was a shadow of true diplomacy, others noted benefits to everyone switching to remote working—for example, several felt they had been able to ‘meet’ virtually with individuals and companies they would never normally have had the chance to meet. Caligiuri et al. [10] suggest that the pandemic offers an ideal time to foster cross-cultural cohesion since everyone, across the world, has a shared experience and a shared ‘enemy’ in COVID-19, and this along with the new opportunities for remote networking mean there could be new opportunities for diplomatic staff during a prolonged crisis such as a pandemic.

Despite the challenges of working from home, most participants appreciated the flexibility it afforded them and did not feel that going back to the office full-time would be helpful. Indeed, most wanted a hybrid model of working in the future, allowing them to work from home or in the office as they pleased. We suggest that individual choice is important here, as different circumstances and needs will mean that some staff may wish to work from home nearly all of the time whilst others would like a roughly 50/50 split and others still may prefer to never work from home. Staff may therefore benefit from measures allowing them autonomy in their return to work, such as allowing them to return at their own pace, allowing them to go to the office just for meetings and then leave again, and offering options for long-term hybrid working. Allowing employees to make their own decisions about remote working would improve perceived job autonomy which in turn is likely to lead to better mental health and better job satisfaction [32]. In order to facilitate a potential hybrid model of working, an organisational ‘flexible working group’ could be set up to share guidance for remote working and tips for working flexibly.

Additionally, good communication is essential during a stressful and ever-evolving situation such as the COVID-19 pandemic, with the need for enhanced communication between managers and employees more important than ever [27]. Scholars have suggested that managers should communicate frequently, clearly and transparently with their staff about both short-term and long-term plans [33–35]; task priorities [36]; expectations of the employee [37, 38]; information regarding how COVID-19 may impact work [39]; organisational goals [38]; and decisions relating to the business continuity plan of the organisation [40]. Managers should also encourage their staff to discuss with them any challenges they are facing [36]. Good communication from managers is likely to improve employees’ experiences of remote working. This is in line with literature which recommends training programmes for homeworkers focused on communication to maximise daily productivity [41].

We acknowledge that participants themselves were conflicted about the overall gains and losses they had experienced working from home during the pandemic, with many reporting both benefits and challenges; this is likely common across occupational groups, and makes it difficult for organisations to know how to respond to conflicting ideas. Remote working experiences are likely to be influenced by employees’ different personalities, circumstances, backgrounds, family situations, work situations, the countries they were living and working in during the pandemic and the leadership in their particular areas. It is clear that there is no one-size-fits-all approach to supporting people with such a range of experiences and needs. While there may be no ‘perfect solution’ to meeting all the various needs of staff, simply asking people what they want and what they think would be helpful for their particular circumstances could be extremely beneficial. As the world begins to return to ‘business as usual’, it is important for organisations to ensure that staff wellbeing and job satisfaction are maintained. If employees are forced to return to face-to-face working when they are not comfortable doing so, this could decrease job satisfaction and morale within the organisation, and could increase turnover intentions. For example, a recent study found that over half of surveyed professionals would rather resign than return to the office full-time [42] and there has been a great deal of news coverage suggesting that ‘forcing’ employees back to the office could result in mass resignations [43, 44]. This is particularly important for diplomatic organisations, given that recent research suggests that the traditional career model of a diplomat (joining the organisation at a young age and spending their entire career there) is beginning to change, with younger cohorts of diplomats no longer considering diplomacy a ‘career for life’ and being much less likely than older cohorts to tolerate the negative aspects of the job and more likely to leave to start a new career if they are not happy with organisational decisions [45].

### Strengths

This is the first study to our knowledge which explores the lived experiences of diplomatic personnel working from home during the COVID-19 pandemic. The study therefore fills a gap in the literature and provides a foundation for future research to build upon. The study supports many of the recommendations identified in previous research on remote working [26, 46] as well as identifies new ones specific to diplomatic personnel. Participants were very varied in terms of their circumstances and pandemic experiences; they had been based in a number of different countries during the pandemic, and represented a wide range of different grades and roles. The participants are therefore representative of a larger percentage of the organisation than they would be if we had, for example, limited our recruitment to a small number of countries, grades or roles. The use of semi-structured interviews allowed for flexibility in data collection, providing participants the opportunity to direct the flow of the interview and ensure they covered the topics they found most relevant or important. Good rapport was built between interviewer and participant, beginning at the initial stages of recruitment with informal, friendly emails to confirm eligibility and arrange interview times and continuing throughout the interviews – this meant that participants felt comfortable to talk about experiences that were very personal to them and therefore helped the interviewer to gain rich data.

### Limitations

Transcripts were independently coded by only one author; ideally, a double-coding process with a second author would help minimise potential bias in coding. However, the other authors read through the list of themes and the quotes chosen to reflect each theme and agreed that the coding and analysis was logical. The sample size was small: although this can be beneficial in terms of making it easier for the interviewer to develop rapport with all participants [47] it can lead to results which are not necessarily representative of the wider population. Despite the relatively small number of participants, the researchers were satisfied that data saturation (the point at which no new themes emerge from the data) [48] was reached. Whilst this suggests that data from 25 employees was sufficient for the analysis, it is unclear how representative these participants may be of FCDO employees in general. There may have been selection bias in that people with particularly strong views may have been more likely to volunteer; their opinions on remote working may therefore be more extreme than those of the average FCDO employee. Additionally, the sample was homogenous, as participants were all diplomatic personnel working for the UK’s Foreign, Commonwealth and Development Office; diplomats representing other countries may have had different experiences. Further, until more research on remote working experiences in the general population is published, it is unclear how well the diplomats’ experiences might generalise to other occupational groups.

Our study’s exclusion criteria meant that staff perceived as potentially experiencing significant distress as a result of the study were excluded from the invite list. This was done for ethical reasons in order to ensure we could protect participants from harm, but it is important to acknowledge that if this had not been one of the exclusion criteria, such participants may have responded differently and we may have identified additional negative effects of working from home.

Although participants were assured of confidentiality and anonymity both before and after their interviews, some may have remained concerned that they may be able to be identified, and so may have deliberately held back information in their responses. Indeed, some participants requested that we not report the specific countries they were based in, which suggests confidentiality is very important to them. It must therefore be considered that some may have downplayed or avoided certain subjects. Finally, we must acknowledge the potential for social desirability bias in participants’ responses; they may have felt uncomfortable providing opinions they perceived to be controversial, or may have wanted to provide responses they predicted the interviewer would want to hear.

### Implications

While all participants in this study were FCDO employees, many of their experiences will be similar to those of other international workers and indeed many other workers in general. The recommendations emerging from this data are therefore likely to be relevant not only to diplomatic organisations but to many other occupational groups.

One key implication of our findings is the importance of flexibility: we suggest organisations should be flexible in terms of allowing staff to choose when and where they work (where possible) and should take into account employees’ individual circumstances and preferences.

Other recommendations include:

- Encourage appropriate work-life balance; line managers should model good practice by ensuring they demonstrate that they maintain a good work-life balance themselves or at the very least strongly encourage their staff to do so
- Encourage time for rest and relaxation at home and discourage responding to work-related calls and emails outside of regular working hours unless absolutely essential
- Remote meetings and social events for teams, to foster a sense of community
- Ensure those in management positions do not pressure staff to put themselves at unnecessary risk during a public health crisis (e.g. do not force them to take part in large gatherings where they would feel unsafe) and consider highlighting a whistle-blowing process
- Line managers to be proactive during a prolonged crisis, checking in on staff and organising virtual wellbeing events or catch-up chats (which should be optional, not mandatory)
- Line managers to be equipped with skills and confidence to speak with staff about their mental wellbeing
- Create a team/organisational atmosphere of ‘overcoming difficulties together’
- Recognise employees’ various unique circumstances and demonstrate flexibility in dealing with them
- Respect childcare situations when defining work arrangements
- Encourage virtual ‘farewell’ events for those leaving old posts during a period of remote working
- Encourage staff to recognise it is common to feel disappointment at the lack of closure if leaving old posts during a period of remote working, and encourage focusing on the positives e.g. reflecting on what went well/considering how the work could be used to inform work in the future.

## Supplementary Information

**Additional file 1**. Examples of quotes illustrating themes and sub-themes.

## Data Availability

The datasets used and/or analysed during the current study are available from the corresponding author on reasonable request.

## Abbreviations

COVID-19/COVID: Novel coronavirus emerging in 2019
FCDO: Foreign, Commonwealth and Development Office
P: Participant
UK: United Kingdom
WHO: World Health Organization
