# Supplementary material for: “In the office nine to five, five days a week… those days are gone”: qualitative exploration of diplomatic personnel’s experiences of remote working during the COVID-19 pandemic
Source: BMC Psychol. 2022 Nov 17;10:272. doi: 10.1186/s40359-022-00970-x (PMC9670069; doi:10.1186/s40359-022-00970-x)
Supplement: Supplementary file 1 — Additional file 1. Examples of quotes illustrating themes and sub-themes. [file 40359_2022_970_MOESM1_ESM.docx]

**Supplementary Appendix 1.**

| **Theme / sub-theme** | **Example quote(s)** |
| --- | --- |
| *Impact of the pandemic on work* | |
| Pre-COVID experiences of remote working | *“I very rarely did it (…) maybe the odd day when you had a report to write or something, but no, on a day-to-day basis absolutely not and really valued actually being with my teams” (P16).*  *“I found the Foreign Office so behind the curve, ‘cause [in previous organisation] we could work remotely from when I joined in 2001, and it’s taken the pandemic for that to be possible in the Foreign Office” (P5).*  *“I was actually already a pioneer for flexible working (…) I worked half the time in the office and half the time from home (…) I was already showing that the work could be done which was actually quite helpful during this scenario because it showed other people that you could move to this remote working quite easily” (P21).* |
| Changes to working style | “A*s an ambassador or any diplomat, your job really entails going out a lot and interfacing with people or going to events and visiting people and places and all of that came to a crashing halt” (P20).* |
| Productivity | *“My productivity, my outputs have just gone through the roof for being able to just get on and focus (…) I find working in an office and with other people frustrating so for me, being able to work at home was (…) brilliant, it meant I could give total focus, no distractions, just crack on and get [project] done” (P10).*  *“I felt like I was much more productive when I was in the office (…) I quite like having almost the pressure of lots of people around me and feeling like I have to kind of really focus (…) I think when I sit in the same room all day staring at the same computer, there's a kind of a boredom element that kicks in” (P3).*  *“Working from home and doing things virtually (…) brought about kind of a whole set of new stresses (…) particularly around kind of how long things would take to get done [due to] inability to have those kind of in-person quick discussions with people” (P9).* |
| Privacy and security | *“Because of the type of work we do I just think it really lends itself to be doing it away from the office, because you have so many sensitive conversations. You were forever having to go from your desk and go and find a room to have a conversation so if you’re just at home doing it, it’s (…) easier in that respect” (P11).*  *“I didn't have access to the right kind of security systems and stuff like that and wasn't able to discuss the kind of sensitive nature of the policy work I was doing with my colleagues on a kind of open line like a Teams call” (P3).* |
| *Relationships with colleagues* | |
| Sense of community | *“It does make you feel more of a part of a bigger network, because you can just connect by Teams which we just didn’t do before” (P19).*  *“One of the benefits of COVID is I’ve had more interaction with my colleagues around the world and around the region than I would have done before” (P22).*  *“Despite this kind of upheaval in terms of remote working and workloads, I was actually getting to meet more people, getting to get a better sense of the network I’d joined and the types of people involved” (P2).*  *“Developing closer friendships with [colleagues] (…) probably partly as a result of things like lessened opportunities to meet people outside of work (…) and also with the travel ban, it meant that for eighteen months we couldn’t have any visitors here so I think that kind of pushed people together a bit more” (P9).* |
| Reduced interaction with colleagues | *“I didn't feel I could get the regular interaction with seniors that I needed when I was working remotely and just kind of wasn't allowing me to progress that piece of work as much as I wanted to” (P3).*  *“I do miss the office as well 'cause of the banter and the seeing everybody” (P11).*  *“If you disagree with something or if you needed to seek more clarity on something and perhaps have kind of more searching, questioning conversations, being able to do that in person (…) feels a lot easier (…) trying to kind of ask these searching questions when you're doing it remotely (…) it’s harder to understand where decisions are being made from and kind of who's directing something when it it's remote” (P9).*  *“It’s been much harder to meet externals and travel, much less opportunity for kind of social things outside of the immediate embassy bubble, which all kind of have a knock-on welfare impact” (P3).* |
| Being away from negative relationships | *“That was the best bit about working from home really, that I was away (…) that helped me because I wasn’t on constant alert about that person, around that person” (P11).* |
| *Benefits of working from home* | |
| Freedom and flexibility | *“Being able to do (…) mini tasks whilst I was working from home (…) meant that I could kind of block out my time more easily both in a work sense but also in a personal sense, like not having to use weekend time to put laundry on or something (…) that made life a lot better. I think that kind of flexibility for me is really good” (P3).*  *“It is nice not to have to commute (…) our working hours were always long and they’ve got slightly longer I’d say, but now at least we don’t have to commute around it” (P1)*  *“I’ve had more flexibility (…) like how I run my day, so particularly around being able to find more time to exercise (…) if you’re kind of working a long day and kind of travelling in, you either didn’t have the time or it was kind of the last thing you wanted to do at the end of a long day” (P9).* |
| New opportunities | *“It’s interesting doing a job where you’re a virtual leader across such a big network, because I’ve not had that experience before and so I’ve had to learn how to do it virtually” (P5).*  *“In terms of my objectives to shine a light on [Oceanic cities] it enabled me to do that with the UK audience in a way that I wouldn’t have been able to do in person (…) I would struggle to get companies to come as far as [Oceanic city] (…) I used it to my advantage because I was able to have engagement with companies (…) and introduce them to opportunities here that I would never have been able to do in person (…) we have been able to do far more than we would do if people were travelling here” (P13).*  *“We’ve seized the opportunity of COVID to renovate the embassy, it was something we were planning on doing anyway, but we’ve been able to do a lot more because people haven’t been physically in the building, so you can obviously rip everything out without having to worry about providing desks for everyone, so that has been a silver lining for us” (P4).* |
| Inclusivity | *“A lot of what we do is networking and a lot of what we do is in person (…) there are people in the team for whom that isn’t comfortable, you know they’re quite introverted and they don’t find it easy (…) so for us to do events online meant that they’re still able to participate but they don’t have that kind of awkwardness where they would perhaps find it a bit intimidating in a physical environment. Being able to do stuff online has enabled them to participate” (P13).*  *“You talk to your colleagues who are autistic, many of whom have found working from home to be an absolute delight and think Teams is amazing (…) for eye contact with people, you know” (P12).* |
| *Challenges of working from home* | |
| Time zones | *“It's not easy when you’re remote working for seven countries and they've got a two-hour time difference ahead of you. So you're up earlier to make sure you're on time with everybody else, and it was a bit stressful” (P6).* |
| Home ergonomics | *“You kind of find yourself sitting at the kitchen table or dining table (…) sitting on a chair not designed for sitting on all day, working at it for eight hours” (P25).*  *“I was tied into a flat where it didn’t really have like a logical place I could that easily and comfortably work from home” (P3).*  *“It became a bit stressful because you're trying to work in the house and do calls and whatever, but you've got the family there and the house is not really (…) equipped to have an office because it was more of a place to go when we're all coming back on holiday” (P6).* |
| Reliance on screens and technology | *“The most used phrase in the vocabulary is ‘I think you might be on mute’. You know, never said those words before in my life, now I say them every day, ‘I think you might be on mute’ (…) I see it as a bit like a séance, ‘is anybody there? Can you see me? Can you hear me? You might be on mute’. It quickly became the new way of working so you kind of quickly had to adapt and get used to it” (P13).* |
| Work-life balance | *“That was quite tough, ‘cause obviously when we’re living on a compound living and working on a compound together and it’s really really intense, so it’s even more important to have that kind of relaxation outside of work which we obviously weren’t able to do” (P16).* |
| *Family* | |
| Improved relationships | *“I got to spend loads of time with my mum (…) before that I was on a posting for three years so obviously only saw her a couple of times a year (…) to actually go and be able to be with Mum for all that time was a real blessing” (P11).* |
| Childcare | *“I didn't know how long it's going to be for so I couldn't put children in nursery (…) I didn't want to uproot them by placing them into a nursery, only to then move them to [Southern African country], so I just did a very big juggle and like hour by hour found childcare whilst working upstairs on my bed, and working [Southern African country] hours” (P18).*  *“Not everyone has a family and the commitments at home and everything that means they can’t give their all to their job (…) not everyone understood the realities of being a working mum, juggling everything (…) this crisis is not (…) one where we go to work, put our all in and we deal with the crisis and then we go home to recuperate before our next shift (…) it genuinely felt like a lot of people hadn't clocked that (…) I wonder how much people actually were supportive and how much people were secretly thinking ‘oh, she's just milking it’” (P21).*  “J*ust muddling through really (…) do work at ridiculous hours of the night to try and catch up and everything and I felt that I was missing out on all the important meetings because they were always happening at times that I couldn't do them” (P21).*  *“Going from (…) the person that normally ran all of this to being someone that was kind of in the background (…) I found that really hard to deal with (…) I felt I had to relegate myself to the background, and I didn't feel I was able to do my job properly” (P21).*  *“My direct manager at the time was also a mum, but with older children. So she understood what I was going through. My director (…) such a lovely guy but totally detached from a working mother’s life because you know he's got a wife at home looks after the children” (P21).* |
| *Moving posts during the pandemic* | |
| Adjusting to new posts | *“I already had quite a good sense of what pre-pandemic in quotes ‘normal’ life was like (…) I knew who everyone was (…) and thereby it means that when working virtually and remotely it's much easier working with those people than had it been with me just starting kind of virtually. I know from others who have started working here in the pandemic how much harder it's been without that kind of foundation of what normal life was like before, having never seen that or been a part of it” (P9).*  *“It’s a leap of faith (…) normally I’m able to come out for four days (…) beforehand to at least see the lay of the land (…) but I wasn’t able to and so the first time I saw my office or came in was the first day I came in for my handover” (P24).*  *“I found it tough moving when I don’t have that support mechanism here, I don’t have friends here, I didn’t know my colleagues here, it’s been hard to make friends because everything shuts (…) I’m going slowly nuts because I can’t get out and meet people in the same way that I used to, so that’s been hard (…) some days I feel it’s quite isolated and lonely” (P5).* |
| Lack of social events | *“I found it really, really isolating (…) obviously when you are abroad and living on your own, you may not have as many connections anyway (…) normally when I’ve arrived at a post you get invited to loads of things, you go to lots of events, if anything you’re almost bombarded or you were in the past with receptions, events to kind of introduce you to your new job and the new country (…) there’s been absolutely none of that, and I personally know absolutely nobody in [Southern European country] apart from people at work or like my immediate neighbours and people in the building, which is really tough” (P16).* |
| Language barriers | *“It was incredibly hard because I didn’t meet my colleagues face-to-face (…) I speak Spanish, but it’s very different speaking Spanish to a person than it is on a Zoom screen (…) you feel a lot more self-conscious” (P1).* |
| Leaving old posts | *“Starting here where maybe you hadn't been able to say goodbye to family, friends 'cause they were (…) in lockdowns at that point or you know, 'cause you haven't really left the house in a few months (…) those kind of things have a much bigger impact” (P3).* |
| *Perceptions and predictions of post-pandemic work* | |
| New views on remote working | *“It’s shown us how good our IT [information technology] is, because we wouldn’t have survived through the pandemic ten years ago or twenty years ago with the IT we had then. It has made us try different ways of working that we might not have ever tried in the past (…) been too busy doing it the traditional way, so I think it’s forced us to look at different ways to do things (…) different ways of working and re-inventing things” (P20).*  *“Today for example I’m working from home and I just told the ambassador yesterday, ‘I’m gonna work from home tomorrow’, and he said yeah of course absolutely fine. You know that IT is tested and we know that it works well for long periods of working from home” (P17).* |
| Benefits of returning to face-to-face working | *“You require the interactions, you require the ability to build rapport and relationships (…) it’s better to have face to face [meetings], you can see that body language and how they’re acting, how they’re talking (…) I think people are adapting, in the future it’s definitely going to be a blended model but I think people are naïve if you think you can completely go away from that sort of working” (P24).* |
| Challenges of returning to face-to-face working | *“It's interesting seeing the press reports about how civil servants are lazy (…) because the government had for years in the Foreign Office (…) been cutting down desks (…) people start to have to work from home, ‘cause there's literally nowhere to work” (P1).*  *“When you do go into the office now isn’t necessarily like what it was before, because people go in maybe a few times a week, you never really know on the day you go in who’s going to be there (…) it’s very quiet in there to the extent where you’re like, if one of the reasons you’re going in is social interaction, you may as well have just stayed at home” (P9).* |
| Hybrid working | *“We would be very happy with the middle ground and for it to stay as a hybrid arrangement for some time if not forever (…) I don’t think I’ve come across anybody who’s keen to come back commuting into the office five days a week and back to the way things were (…) I think there’s a real appetite for hybrid working to continue well into the future” (P20).*  *“I think individual managers, ambassadors, directors will approach it in their own way (…) some (…) would definitely want to try and get people back to the office as much as possible, and there will be (…) pushback because of the benefits and so I think there might be a kind of a bit of a rocky period as we're coming out of the pandemic (…) where people are not happy to go back, whether it's because of health reasons or just they've discovered how much more effective they can be working from home (…) there might be a bit of friction with managers who really want to get them back to the office, but I think it will iron itself out (…) I think the changes will continue, but there will be a bit of a difficult transition period” (P16).* |
| Considerations for the future | *“I think it’s a massive benefit that we’ve moved towards hybrid working but I don’t think we’ve quite yet accepted that there can be a lot of flexibility within that, and it’s not a kind of one size fits all” (P16).*  *“Whereas we used to go into the office for a full day, now maybe you go into the office for part of the day when you have in-person meetings and you may leave and go home after that and finish working at home” (P9).*  *“The ambassador sort of mandated he wanted people back in three days a week in the office (…) that didn’t make sense to me (…) it would maybe make a lot more sense to maybe just go into the office one day, have all your meetings with the teams there, but then kind of spend the rest of the week [at home]” (P16).* |
